# Repetitive transcranial magnetic stimulation increases synaptic plasticity of cortical axons in the APP/PS1 amyloidosis mouse model

**DOI:** 10.1117/1.NPh.12.S1.S14613

**Published:** 2025-05-28

**Authors:** Barbora Fulopova, William Bennett, Alison J. Canty

**Affiliations:** a The University of Queensland, The Queensland Brain Institute, St. Lucia, Queensland, Australia; b University of Tasmania, Wicking Dementia Research and Education Centre, Hobart, Tasmania, Australia

**Keywords:** live imaging, two-photon microscopy, axon, bouton, dementia, synaptic plasticity

## Abstract

**Significance:**

Growing evidence highlights the therapeutic potential of repetitive transcranial magnetic stimulation (rTMS) in diseases causing dementias such as Alzheimer’s disease (AD). However, individual responses to rTMS are variable, and its underlying neural mechanisms are not fully understood.

**Aim:**

As synaptic dysfunction is one of the key mechanisms associated with cognitive deficits in dementia, we investigated the effect of rTMS on cortical synapses using an APP/PS1 amyloidosis mouse model of AD crossed with fluorescent reporters linked to the Thy-1 promoter.

**Approach:**

Using *in vivo* two-photon imaging, we characterized the plasticity of excitatory *terminaux* (TB) and *en passant* (EPB) axonal boutons at 48-h intervals for 8 days on either side of a single session of rTMS.

**Results:**

We found both types of axonal boutons preserved the overall number of their synaptic outputs in wild type (WT) and APP/PS1 groups, pre- and post-stimulation. Both synapse types also showed a significantly reduced dynamic fraction in APP/PS1 compared with WT axons pre-stimulation. Following stimulation, the TB, but not EPB, dynamic fraction increased in both WT and APP/PS1 groups.

**Conclusions:**

This suggests possible mechanisms of rTMS action that are cell type-specific and, together with previous findings of improved functional performance, present a potential clinical avenue for rTMS in the management of AD.

## 1 Introduction

Alzheimer’s disease (AD) is a terminal, neurodegenerative, dementia-causing disease characterized by progressive cognitive and behavioral decline. To date, there is no effective cure and limited treatment options for symptom management. Repetitive transcranial magnetic stimulation (rTMS) is a localized, noninvasive form of brain stimulation emerging as a potential therapeutic option for alleviating cognitive symptoms experienced in dementia-causing diseases such as AD.^1^ However, individual response rates can be variable, and the underlying neural mechanisms of rTMS in AD have not yet been fully described.^2^ In a healthy nervous system, biological consequences of rTMS are commonly linked to neuroplasticity-like mechanisms. In particular, rTMS delivered as intermittent theta burst stimulation (iTBS) is associated with an increase in neural excitability of stimulated cortical areas that outlasts the duration of the stimulation session,^3^ long-term neuroplastic cortical reorganization that underpins changes in learning and cognition,^4^^–^^6^ increase in functional brain connectivity,^7^ and synaptic facilitation at glutamatergic synapses via N-methyl-d-aspartate receptor activation and modulation of intracellular calcium signaling.^8^^–^^10^

Synaptic dysfunction is a key pathological feature associated with cognitive deficits observed in AD,^11^^,^^12^ yet our understanding of any synaptic impact of iTBS in the presence of AD pathology is limited. In the APP23/PS45 double transgenic model of AD, low-intensity rTMS was found to improve memory performance, reduce amyloid-beta pathology, and ameliorate impairment of hippocampal synaptic plasticity.^13^ In a vascular dementia model, rTMS was associated with improved learning, increased expression of BDNF, and higher density of cholinergic neurons in the hippocampus,^14^ as well as upregulation of synaptic proteins^15^ and downregulated expression of pro-apoptotic proteins.^16^ More recently, iTBS was found to attenuate cognitive decline and amyloid pathology development and preserve mitochondrial function in APP/PS1 mice.^17^ Taken together, rTMS delivered in a nervous system marked by amyloid pathology induces cellular and behavioral changes comparable to those observed in a pathology-free brain. Here, we examined how iTBS affects cortical synapses in the adult APP/PS1 amyloidosis mouse model of AD, compared with their wild-type counterparts. We used longitudinal *in vivo* two-photon imaging to assess dynamic adaptations at the excitatory neural synapse following low-intensity (LI) iTBS delivered via a rodent-specific transcranial magnetic stimulation coil.^18^ Previously, we have shown that a single session of LI-iTBS is followed by a transient increase in the loss of dendritic spines in the primary motor cortex of healthy adult mice.^19^ Here, we focused on presynaptic axonal boutons. Much like postsynaptic dendritic spines, axonal boutons are capable of neuroplastic adaptation; however, our understanding of their adaptive properties is limited. Previous findings suggest axonal boutons are dynamic structures that can rapidly respond to changes in environmental demands.^20^^–^^23^ Any changes in the number of boutons and/or their postsynaptic targets can have profound effects on the functional outcome of the local microcircuitry. Here, we quantified changes in density and dynamics (turnover, gains, and losses over time) across two types of cortical boutons—*en passant boutons* (EPBs) that appear as swellings along the main axon shaft and *terminaux boutons* (TBs) that protrude sideways from the main axon shaft.^24^ We found that both types of boutons show deficits in turnover but not in their density in the APP/PS1 mice. Following the stimulation, TBs but not EPBs transiently increased their turnover in both the APP/PS1 and wild-type mice. In the APP/PS1, the increase in TB bouton dynamics rescued their pre-stimulation deficit. Cumulatively, these results suggest that LI-iTBS promotes dynamic restructuring of presynaptic excitatory synapses in the presence of amyloid pathology and offers support for clinical application of rTMS to noninvasively manage synaptic dysregulation associated with AD.

## 2 Materials and Methods

### 2.1 Experimental Sample

All experiments were performed in 10- to 13-month-old male mice. C57BL/6 mice expressing a green fluorescent protein under the Thy1 promoter (Thy1-GFP-M; IMSR_JAX:007788) were used as a control group (WT-GFP; n=8 animals; 25 axonal segments with 757 boutons across combined length of 5.6 mm). The amyloidosis group (APP-GFP; n=6 animals, 25 axonal segments with 466 boutons across combined length of 4.2 mm) consisted of Thy1-GFP-M mice bred with APP/PS1 mice [mutant human amyloid precursor protein (Mo/HuAPP695swe, mutant human presenilin 1; MMRRC_034832-JAX)] on the same background strain C57BL/6.^25^ All mice were co-housed, 4 to 5 mice per Optimice cage (a caging system that contains no noise or vibration from motors or blowers, providing a low-stress environment for the animals), *ad libitum* access to food and water. All experiments were performed in accordance with the Australian Code of Practice for the Care and Use of Animals for Scientific Purposes and approved by the University of Tasmania Animal Ethics Committee (A17371).

### 2.2 Cranial Window Implantation

A previously established craniotomy protocol was adapted.^26^^,^^27^ Briefly, the mice were administered analgesic Temgesic (Buprenorphine; 0.1  mg/kg) 30 min prior to the surgery, then anesthetized with isoflurane (5% induction, 2.5% maintenance in 100% oxygen), and placed in a stereotaxic frame. Local anesthetic bupivacaine (5  mg/kg) was subcutaneously applied to the scalp area before the initial incision. The right parietal skull bone overlaying the somatosensory cortex region SS1 was removed using a high-speed fine-motorized drill, leaving the exposed dura intact. This area was chosen for imaging due to ease of access (directly underneath the right parietal bone) and for consistency with previously published reports of synaptic dynamics in cortical axons.^20^^,^^28^^,^^29^ Sterile cortical buffer (125-mM NaCl, 5-mM KCl, 10-mM glucose, 10-mM HEPES, 2-mM ${\mathrm{CaCl}}_{2}$, 2-mM ${\mathrm{MgSO}}_{4}$, pH 7.4) was applied regularly during drilling to cool the site and wash away any debris. Dexamethasone (4  mg/mL) was applied topically to the dura to suppress any meningeal inflammatory response, and immediately after, a 5-mm glass coverslip was placed over the craniotomy and secured with instant adhesive gel (Loctite 454) and sealed in place with dental acrylic. Animals were allowed a 3-week rest period before commencing imaging.

### 2.3 *In Vivo* Imaging and Quantification of Axonal Bouton Dynamics

**Fig. 1:**
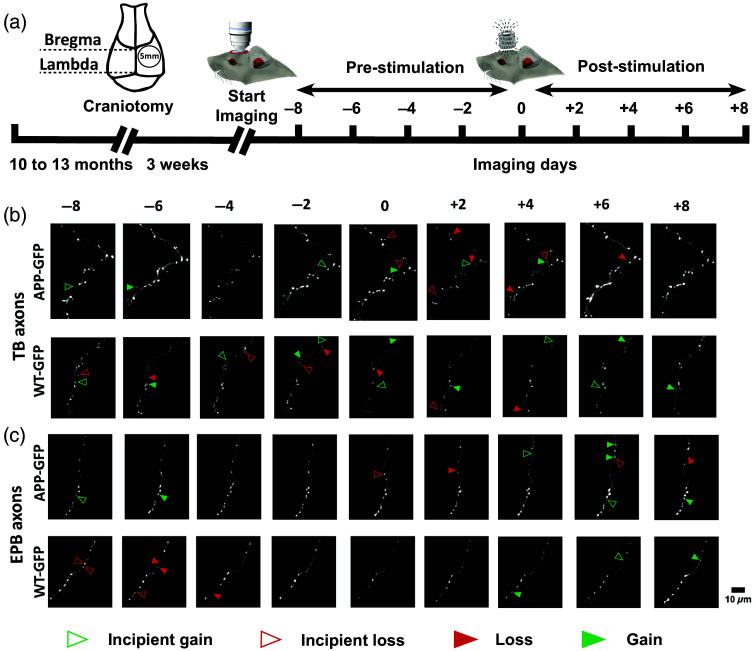
Experimental methodology. (a) Experimental timeline: craniotomy performed in 10- to 13-month-old mice, imaging commenced after a 3-week recovery period. *In vivo* imaging was conducted at 2-day intervals, with one round of stimulation shortly after day 0 imaging. Examples of repeated imaging of axonal segments rich in EPBs (c) and TBs (b) shown on maximum projection images (WT-GFP and APP-GFP). Arrowheads indicate sites of gains/losses of boutons between consecutive sessions. (WT–GFP—wild type crossed onto Thy1-GFP background; APP-GFP—APP/PS1 crossed on Thy1-GFP background; EPB—*en passant bouton*, TB—*terminaux bouton*).

Two-photon imaging was performed using a custom-built set up as previously described.^27^ Briefly, an upright laser scanning microscope (Scientifica, Uckfield, United Kingdom) was equipped with galvo–galvo mirrors, a high-sensitivity GaAsP nondescanned photomultiplier detector (Hamamatsu) and ×20 water immersion objective (NA 1.0, Zeiss Plan-Apo, Oberkochen, Germany). The excitation source was a femtosecond-pulsed Ti-sapphire laser with group velocity dispersion pre-compensation (Mai Tai DeepSee, Spectra-Physics, Milpitas, California) and tuned to 910 nm for GFP excitation. Laser power attenuation was mediated via a Pockels cell, controlled by Conoptics 302RM differential amplifier. Typically, we delivered 20 to 90 mW power at the back aperture. The live imaging was conducted under isoflurane anesthesia (∼2% in 100% oxygen). During and immediately after the anesthesia, we monitored the animals closely to avoid and manage any complications. The imaging stage was equipped with a heated pad set to 37°C, to maintain stable body temperature during the imaging session. Prior to the onset of imaging, animals were subcutaneously administered a 0.5-mL saline to ensure hydration. The typical length of anesthesia was ∼60  min. During this time, the animals were regularly monitored (∼10-min intervals) for any signs of change in respiratory rate (∼70 breaths per minute [BPM]), withdrawal response, and absence of whisking. All anesthesia levels and exposure times were consistent between WT-GFP and APP-GFP, and we did not observe any differences in the monitored physiological parameters between the groups. The experimental timeline consisted of nine imaging sessions conducted at 48-h intervals [Fig. 1(a)]. Images were acquired using ScanImage 3.8.1 software in MATLAB.^30^ An EPB bouton was considered present if it was visible across at least two consecutive z-planes with intensity at least twice that of the axonal backbone [Fig. 1(c)]. A TB was considered present if it was visible across at least two consecutive z-planes and protruding from the axonal backbone at least 1  μm in at least one imaging session and lost at the time point when the protrusion measured less than 0.4  μm [Fig. 1(b)]. Densities of boutons were calculated per axonal length (N total/length; min axonal length 100  μm), and the turnover rate was calculated as a proportion of gains and losses between two consecutive sessions $\left[(N\;{\text{losses}}_{\text{at session}\;B}+N{\;\text{gains}}_{\text{at session}\;B})/(N\;{\text{total}}_{\text{at session}\;A}+N\;{\text{total}}_{\text{at session}\;B})\right]$. The same calculation was performed for the assessment of gains and losses between two consecutive sessions ($N\;{\text{losses}}_{\text{at session}\;B}$ or $N{\;\text{gains}}_{\text{at session}\;B}$ / ($N\;{\text{total}}_{\text{at session}\;A}+N\;{\text{total}}_{\text{at session}\;B}$). To account for possible differential effects of the stimulation based on the position of the axonal processes within the magnetic field, we also recorded the depth from the cortical surface and the orientation of the axonal projections. No effects of depth or direction were detected in our dataset (Figs. S1 and S2 in the Supplementary Material).

### 2.4 Brain Stimulation Protocol

LI-iTBS was delivered directly over the craniotomy site of awake mice using a custom rodent-specific coil as previously described.^18^ The 8-mm diameter coil induced a peak magnetic field of 0.12T at the base of the coil, with maximal field strength typically observed within ∼2-mm z-axis and ∼4-mm xy-axis, which is well within the imaged cortical area.^18^ During stimulation, the coil was held ∼1  mm above the craniotomy. To ensure consistent delivery of the stimulation, mice were gently restrained in a restraint bag with a breathing hole (Able Scientific, Western Australia, Australia). To control for the possible effect of the restraint, the animals were habituated to the protocol during initial screening sessions and were then placed in the restraint bag after each imaging session, to ensure that the LI-iTBS stimulation was the only novel intervention at the time of the stimulation. A total of 600 monophasic pulses of LI-iTBS were delivered to awake mice (triplet pulses at 50 Hz, repeated at 5 Hz for 2-s, 8-s inter-train interval),^8^ lasting 190 s. Mice were observed during and directly after stimulation and displayed no behavioral changes.

### 2.5 Statistical Analysis

All statistical analyses were performed using IBM SPSS software. For bouton densities, the appropriate ANOVA models and t-tests were used, the significance was set at alpha 0.05, and the effect sizes expressed as partial eta squared. Significant main effects and interactions were followed up with simple main effect tests. Data were assessed for meeting the assumptions of ANOVA and t-test prior to analysis. Where appropriate, the equality of covariance matrices was tested using Box’s M. The turnover, gains, and losses data were analyzed using appropriate nonparametric tests. All multiple comparisons were interpreted with respect to Bonferroni adjusted alpha. Graphs were prepared with GraphPad Prism, and figures with images were prepared using Fiji and Adobe suite.

## 3 Results

### 3.1 Pre-stimulation TB and EPB Density is Stable and Comparable in WT-GFP and APP-GFP Axons

**Fig. 2:**
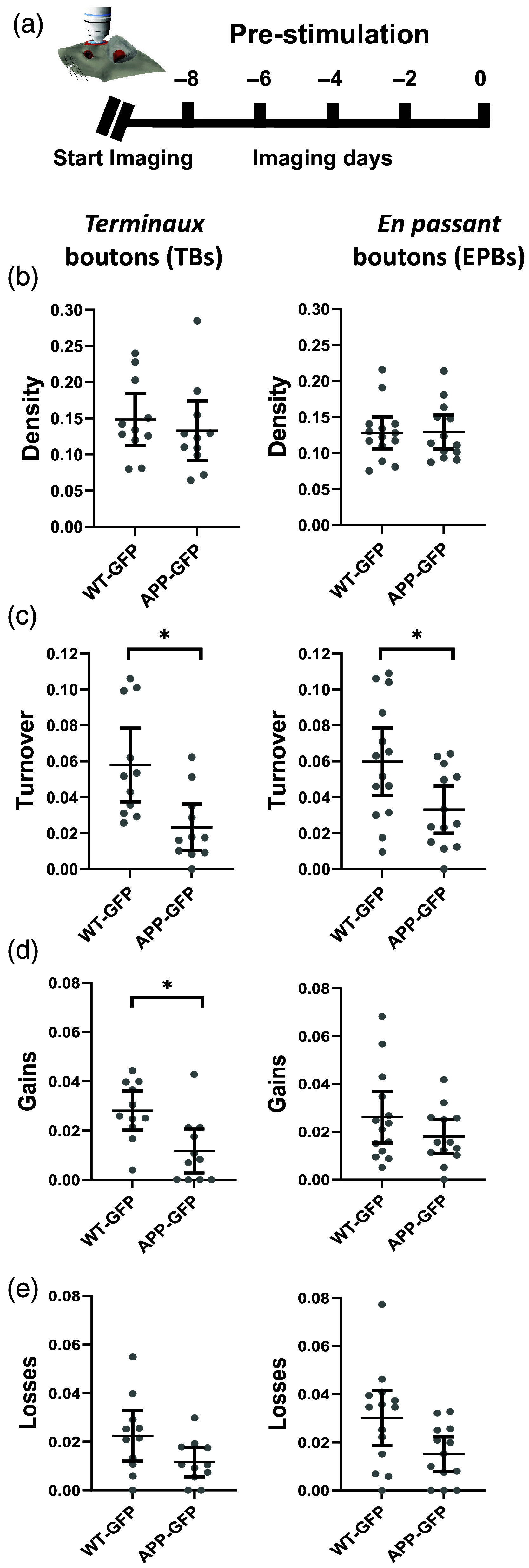
Baseline pre-stimulation properties of axonal *en passant* and *terminaux* boutons. (a) Experimental timeline. There were no significant differences between individual imaging days within each group (see also Fig. 3). Pre-stimulation data for each axon were averaged as a single pre-stimulation mean value. (b) No significant differences in the average density of TBs and EPBs between WT-GFP and APP-GFP. (c) Turnover (expressed as the combined proportion of gains and losses) was significantly lower in the APP-GFP group compared to the WT-GFP for both TBs and EPBs. (d) and (e) The proportion of gains and losses was decreased in APP-GFP TBs and EPBs, but only reached significance for TB gains. (WT–GFP—wild type animal crossed onto Thy1-GFP background; APP-GFP—APP/PS1 animal crossed on Thy1-GFP background, bars are 95% confidence intervals; *statistically significant difference).

To gain an understanding of the innate dynamics of axonal boutons, we first considered their characteristics across the baseline imaging sessions. We analyzed synaptic density across five pre-stimulation sessions [experimental days −8 to 0; Fig. 2(a)] using mixed ANOVA. There was no significant difference in the density of TBs across the five baseline pre-stimulation sessions ($F_{\left(2.1,36.3\right)}=0.77$; p=0.48), and no significant imaging day ∗ genotype interaction ($F_{\left(2.1,36.3\right)}=1.2$; p=0.316). There was also no significant difference detected between the WT-GFP and APP-GFP genotypes [$F_{\left(1,17\right)}=0.6$; p=0.448. Fig. 2(b) right].

Similarly, there were no significant differences observed in the EPB synapses between imaging sessions ($F_{\left(1.85,27.68\right)}=0.26$; p=0.754), imaging day ∗ genotype interaction ($F_{\left(1.85,27.68\right)}=0.22$; p=0.788), nor any differences between the WT-GFP and APP-GFP groups ($F_{\left(1,15\right)}=0.04$; p=0.842). These results suggested an overall stable baseline density of both TBs and EPBs across the pre-stimulation imaging period, with no differences in density between boutons subtypes nor between WT-GFP and APP-GFP groups [Fig. 2(b) left].

### 3.2 Pre-stimulation Turnover of TBs and EPBs is Decreased in APP-GFP Axons

**Fig. 3:**
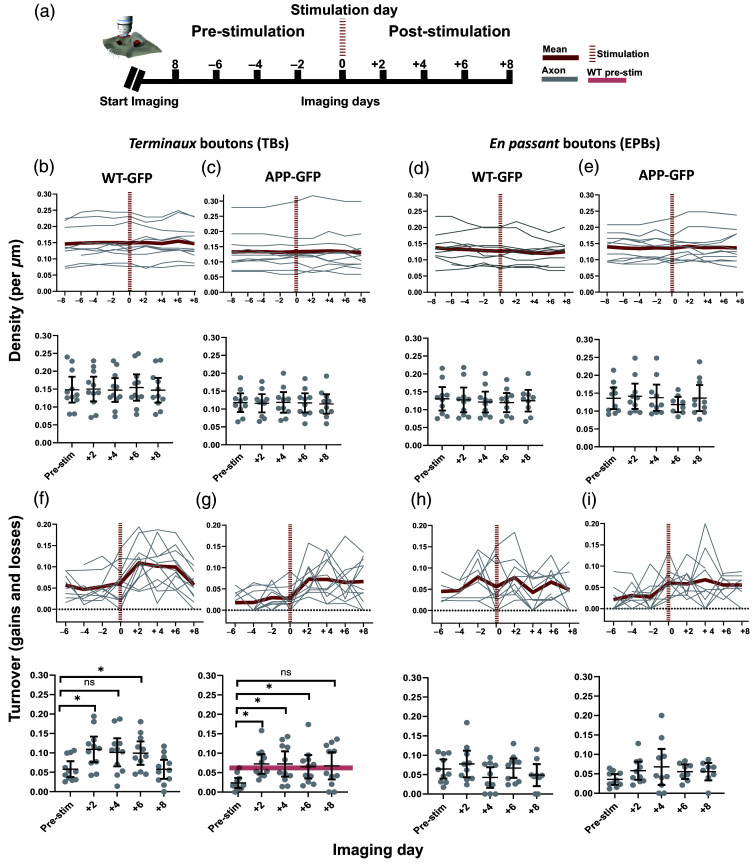
Synaptic dynamics of *en passant* and *terminaux* boutons after LI-iTBS. (a) Experimental timeline. LI-iTBS did not alter the density of TBs or EPBs in neither WT-GFP (b) and (d) or APP-GFP (c) and (e) groups. LI-iTBS significantly increased TB turnover in WT-GFP (f) and APP-GFP (g) groups. In the APP-PS1 group (g), the post-stimulation increase in turnover reached the WT-GFP pre-stimulation baseline (faded line, lower panel). LI-iTBS did not significantly change turnover in the EPBs in either the WT-GFP (h) or APP-GFP (i) groups. (Line graphs show data for individual axons, and point-spread graphs immediately below show corresponding means with 95% confidence intervals; *statistically significant difference; ns—non significant after Bonferroni adjustment; WT–GFP—wild type animal crossed onto Thy1-GFP background; APP-GFP—APP/PS1 animal crossed on Thy1-GFP background.)

Next, we considered a dynamic turnover of the boutons across all baseline pre-stimulation sessions. Within each group, there was no significant difference in synaptic turnover, gains or losses, over the 8 days of imaging pre-stimulation [Table 1, Figs. 3(f)–3(i)], suggesting stable intrinsic plasticity of both types of axonal boutons.

**Table 1:** Outcomes of Friedman ANOVA comparisons of the axonal bouton turnover across the five pre-stimulation days. ($\chi^{2}$—chi-square statistic; p—two-tailed significance value).

Test	Bouton	Turnover		Gain		Loss	
	Type	$\chi^{2}$	p	$\chi^{2}$	p	$\chi^{2}$	p
Friedman ANOVA	TB	2.73	0.44	1.72	0.63	7.26	0.06
WT-GFP	EPB	6.08	0.11	1.38	0.71	3.15	0.37
Friedman ANOVA	TB	5.21	0.16	4.18	0.24	3.34	0.34
APP-GFP	EPB	7.99	0.5	3.65	0.3	5.01	0.17

Therefore, data from the five pre-stimulation imaging sessions were averaged into a single pre-stimulation value for each measure (gains, losses, turnover) for all TB and all EPB axons, and compared between WT-GFP and APP-GFP groups using the Mann Whitney U test. For TB axons, the APP-GFP group (M rank=7.55) had significantly lower baseline pre-stimulation turnover of TBs compared with the WT-GFP group [U=17; p=0.003; $\eta^{2}=0.38$; M rank=14.45; Fig. 2(c) left]. In addition, significantly fewer gains were present in APP-GFP [U=19, p=0.006; $\eta^{2}=0.355$; M rank=7.73; Fig. 2(d)] compared with the WT-GFP group (M rank=15.27). Although there was also a trend toward fewer losses in the APP-GFP group (M rank=8.82) compared with the WT-GFP group (M rank=14.18), this difference was not statistically significant [U=31; p=0.056; Fig. 2(e)].

Similarly, EPB axons in the APP-GFP group had significantly lower turnover [U=47; p=0.03; $\eta^{2}=0.16$; M rank=10.62; Fig. 2(c) right] compared with the WT-GFP group (M rank=17.14). Although there was a trend toward both lower gains and lower losses in the APP-GFP group, this was not significant [gains U=35.5; p=0.27; losses U=27; p=0.08; Figs. 2(d) and 2(e) right]. Taken together, across both bouton subtypes, the combined reduction of gains and losses was represented as an overall decrease in the dynamic fraction of axonal boutons in the APP-GFP amyloidosis group, whereas the overall number of boutons (density) remained unchanged.

### 3.3 LI-iTBS Does Not Alter Bouton Density in WT-GFP or APP-GFP Axons

To understand how/if the previously established bouton dynamics are affected by iTBS, we compared the baseline pre-stimulation to the post-stimulation characteristics. To assess changes in the synaptic density of TBs after stimulation, we compared the averaged pre-stimulation density to +2, +4, +6, and +8 days post-stimulation [Fig. 3(a)]. Using mixed ANOVA analysis, we found no significant imaging day ∗ genotype interaction ($F_{\left(4,80\right)}=0.78$; p=0.541), no significant difference between the density across the imaging sessions ($F_{\left(4,80\right)}=1.01$; p=0.408), and no significant difference in density of TBs between the WT-GFP and APP-GFP groups [$F_{\left(1,20\right)}=0.40$; p=0.535; Figs. 3(b) and 3(c)]. Similarly, in the EPB bouton population, we saw no imaging day ∗ genotype interaction ($F_{\left(2.2,36.83\right)}=1.27$; p=0.3), no significant difference between the density across the imaging sessions ($F_{\left(2.2,36.83\right)}=1.35$; p=0.27), and no significant difference in density of EPBs between the WT-GFP and APP-GFP groups [$F_{\left(1,17\right)}=0.19$; p=0.67; Figs. 3(d) and 3(e)]. This suggests that post-stimulation, the imaged axons maintained an overall stable number of synaptic outputs irrespective of the type of the axonal boutons (TBs or EPBs) or the genotype [WT-GFP or APP-GFP; Figs. 3(b)–3(e)].

### 3.4 LI-iTBS Increases the Dynamic Turnover of TBs But Not EPBs

Next, we considered the effect of stimulation on the dynamic properties of axonal boutons, comparing the averaged pre-stimulation measures of turnover, gains, and losses to +2, +4, +6, and +8 days post-simulation. For EPB boutons, we saw no significant effect of the stimulation in either WT-GFP or APP-GFP groups [Table 2; Figs. 3(h) and 3(i)].

**Table 2:** Wilcoxon signed-ranked p-values. Comparison of mean turnover of EPBs between pre-stimulation and at 2, 4, 6, and 8 days post-stimulation.

Wilcoxon signed-rank p-value for EPBs	+2 days	+4 days	+6 days	+8 days
WT-GFP	0.33	0.07	0.96	0.44
APP-GFP	0.22	0.11	0.21	0.12

For TBs of WT-GFP axons, we saw a significant increase in post-stimulation turnover at +2 and +6 days post-stimulation (Z=−2.67, p=0.008; Z=−2.93, p=0.003, respectively). Although we saw a trend for increased turnover at +4 days, this was not significant after adjusting for multiple comparisons (Z=−2.31, p=0.021). By +8 days, turnover had returned to be within pre-stimulation levels [Z=0.01, p=1; Fig. 3(f)], suggesting that the post-stimulation increase in turnover was transient.

For TBs of APP-GFP axons, we detected a significant increase in turnover at +2, +4, and +6 days post-stimulation (Z=−2.85, p=0.004; Z=−2.85, p=0.004; Z=−2.67, p=0.008, respectively). There was a trend toward increased turnover on day 8; however, this fell short of significance after adjusting for multiple comparisons (Z=−2.31, p=0.021). Given that Bonferroni adjustment is a somewhat conservative correction for inflated type 1 error in multiple comparisons with small sample sizes,^31^ it is possible that the trends in increased turnover in WT-GFP at day +4 and APP-GFP at day +8 are biologically meaningful and could be considered when interpreting the findings [Figs. 3(f) and 3(g)].

### 3.5 Combined Changes in Gains and Losses are Necessary to Drive Increased TB Dynamics Post-Stimulation

**Fig. 4:**
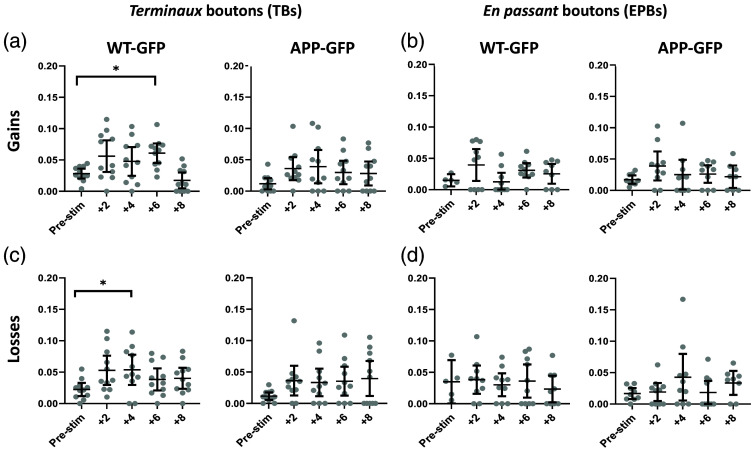
Gains and losses of *terminaux* and *en passant* boutons post-stimulation. (a) and (c) A trend towards increased gains and losses of TBs post-simulation in both WT-GFP and APP-GFP groups. Statistical significance was only observed in WT-GFP gains at day +6, and WT-GFP losses at day +4. (b) and (d) No changes in EPBs gains or losses post-stimulation. (WT–GFP—wild type animal crossed onto Thy1-GFP background; APP-GFP—APP/PS1 animal crossed on Thy1-GFP background, error bars are 95% confidence intervals; *statistically significant difference).

Next, we assessed the individual contribution of synaptic gains and losses to the overall increase in turnover of TBs post-stimulation. In WT-GFP axons, there was a significant increase in gains +6 days post-stimulation (Z=−2.85, p=0.004), with no significant differences detected at +2, +4, or +8 days [p=0.041, p=0.182, p=0.033, respectively; Fig. 4(a) left]. There was a significant increase in losses at +4 days post-stimulation (Z=−2.56, p=0.009), with no differences detected at +2, +6, or +8 days post-stimulation [p=0.041, p=0.041, p=0.110, respectively, Fig. 4(c) left]. In the APP-GFP group, despite trends for increases in both gains and losses across all post-stimulation timepoints, Bonferroni adjusted Wilcoxon signed-rank test indicated no significant difference at +2, +4, +6, or +8 days in either gains (p=0.028, p=0.051, p=0.037, p=0.074, respectively) or losses [p=0.022, p=0.017, p=0.028, p=0.074, respectively; Figs. 4(a) and 4(c) right]. There were no significant changes observed in either gains or losses in EPB synapses in either WT-GFP or APP-GFP groups [Figs. 4(b) and 4(d)]. Taken together, these results suggest that the increase in turnover of TBs was driven by cumulative changes in gains and losses, rather than either of the events preferentially.

## 4 Discussion and Conclusion

The ability of rTMS to noninvasively perturb activity of the central nervous system is clinically pertinent across various neuropsychiatric and neurodegenerative conditions, including the management and/or treatment of Alzheimer’s disease. Here, we report evidence that LI-iTBS promotes structural reorganization of cortical axonal boutons and rescues observed deficits in *terminaux* but not *en passant* bouton dynamics in cortical axons of the APP/PS1 mouse model of amyloidosis.

In APP-GFP cortical axons, both TBs and EPBs showed reduced structural reorganization (turnover), but comparable bouton densities with WT-GFP controls. These findings are largely consistent with other reports of divergent properties of the presynaptic compartment studied *in vivo* in preclinical AD models.^22^^,^^27^^,^^32^ The decrease of bouton dynamics in the APP-GFP, however, is in contrast to previously reported increases in turnover observed in the vicinity of amyloid plaques in the same model at 3 to 4 months of age.^22^ This is likely attributable to differences in pathological stage at the time of imaging. Pathology development in APP/PS1 follows a well-described trajectory of initial rapid onset at 3 months of age that plateaus around 12 months,^25^^,^^33^ and the neurodegenerative microenvironment in the vicinity of amyloid plaques is commonly associated with a host of axonopathies particularly in early stages of the disease development.^34^^,^^35^ In our study of 10- to 13-month mice, amyloid plaque pathology is more mature, akin to human pathology around the time of AD diagnosis, and it is reasonable to assume that surviving cortical axons may have already undergone homeostatic adaptation to the surrounding pathology.

LI-iTBS boosted the turnover of TBs, but not EPBs, in both the WT-GFP and APP-GFP groups, most noticeably at 48-h post-stimulation, with an 88% increase above baseline in WT-GFP, and 213% increase in APP-GFP axons. In the APP-GFP group, this increase reached pre-stimulation turnover levels observed in WT-GFP mice, essentially rescuing the reduced bouton dynamics observed pre-stimulation. The canonical understanding of the anatomical distinction between EPBs and TBs suggests cell type-specific origins particularly pertaining to connectivity. Axons rich in EPBs are more prevalent, and typically associated with long-range projecting axonal segments, and TB-rich axonal segments are often found in local microcircuits at collateral branches.^36^ The TB-rich excitatory axons present in the superficial layers of the cortex that were sampled here resemble those belonging to principal excitatory neurons originating in the cortical layer 6.^24^^,^^37^ Apart from their subcortical output^38^^,^^39^ layer 6 originating excitatory neurons also form ipsilateral interlaminar connections within functionally distinct cortical regions.^40^^,^^41^ The TBs found on these superficially projecting collaterals are typically found to be more plastic than EPBs when imaged over longer time periods.^20^^,^^21^^,^^24^ It is therefore possible that the differential effects of LI-iTBS on the two classes of axonal boutons we observed here are reflective of cell type-specific effects of the stimulation. It remains to be determined if this effect is driven by LI-iTBS influencing the activity of the excitatory principal cells directly, or by acting on the local inhibitory modulators.^42^^,^^43^

To our knowledge, this is the first study to provide evidence for the involvement of pre-synaptic boutons in the cortical axon response to rTMS *in vivo*, in a pathology-free, healthy nervous system, as well as in a nervous system marked by the presence of pronounced amyloidosis associated with dementias. In both conditions, following rTMS, we observed an increase in reorganization of boutons in an axon-type specific manner, suggesting targeted involvement of local microcircuitry as opposed to the indiscriminate effect of exposure to the magnetic field. Given the established link between synaptic dysfunction and cognitive impairment in dementia, the acute rescue of deficits in the pre-synaptic dynamic in the APP/PS1 model observed here provides a potential target for a disease-modifying intervention and/or symptom management aimed at delaying disease progression and enhancing quality of life for people living with dementia. Although some progress has been made in targeting AD pathology pharmacologically, attempts at finding a drug that would arrest or reverse the severe memory impairment experienced by people living with AD remain unsuccessful. Neuromodulation by rTMS has been successfully applied in other neurodegenerative and neuropsychiatric conditions and can be implemented during a broad therapeutic window, offering a powerful alternative and/or addition to currently employed AD management strategies.

### 4.1 Limitations and Future Directions

Whether the effects observed here are reflective of the regional response to rTMS or whether the response of TBs and EPBs differs broadly across the different brain regions should be confirmed by future experiments. In addition, future studies should aim at addressing the active physiological responses to rTMS by measuring real-time neuronal activity in awake behaving animals (i.e. performing a skilled learning or other cognitive paradigms). This would particularly be important to study with repeated stimulation sessions that are more closely reflecting the clinical application of rTMS.

## Supplementary Material

10.1117/1.NPh.12.S1.S14613.s01

## Data Availability

The data that support the findings of this study are available from the corresponding author, upon reasonable request.
