# Supplementary material for: Repetitive transcranial magnetic stimulation increases synaptic plasticity of cortical axons in the APP/PS1 amyloidosis mouse model
Source: Neurophotonics. 2025 May 28;12(Suppl 1):S14613. doi: 10.1117/1.NPh.12.S1.S14613 (PMC12119023; doi:10.1117/1.NPh.12.S1.S14613)
Supplement: Supplementary file 1 [file NPh_012_S14613_SD001.pdf]

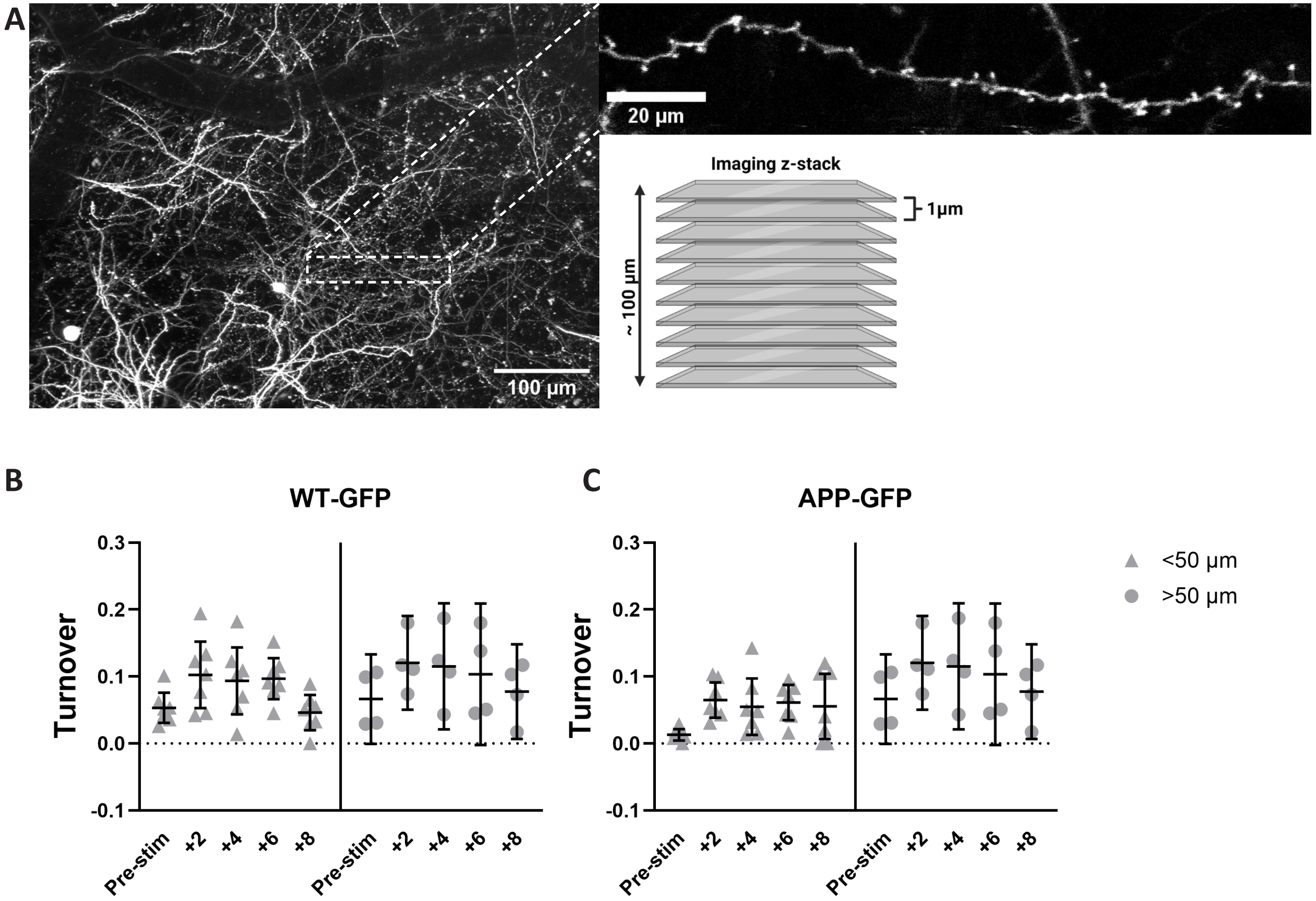

A
20 μm
100 μm
Imaging z-stack
1μm
~ 100 μm
B
WT-GFP
C
APP-GFP
Turnover
0.3
0.2
0.1
0.0
-0.1
Pre-stim
+2
+4
+6
+8
<50 μm
>50 μm

Supplementary figure 1. Depth of imaged axons did not influence turnover of terminaux boutons. **A)** A representative example of cortical area the axons were sampled from, showing 2x3 montage of z-stacks (left) and zoomed in image of a TB axon (right top) located ~60 µm deep in the z-stack. Imaging was done in a grid-like pattern. Approximately ~100 µm z-stacks were acquired for each x,y coordinate in 1µm z-steps (right bottom illustration), starting from the first z-plane with visible neuronal processes. **B&C)** We classified imaged axons into 2 categories based on their depth within the upper layers of labelled neuropil (within or below 50 µm). We saw no trends suggesting differences in turnover based on the depth of axons. (WT–GFP - wild type animal crossed onto Thy1-GFP background; APP-GFP – APP/PS1 animal crossed on Thy1-GFP background, error bars are 95% confidence intervals around the mean).

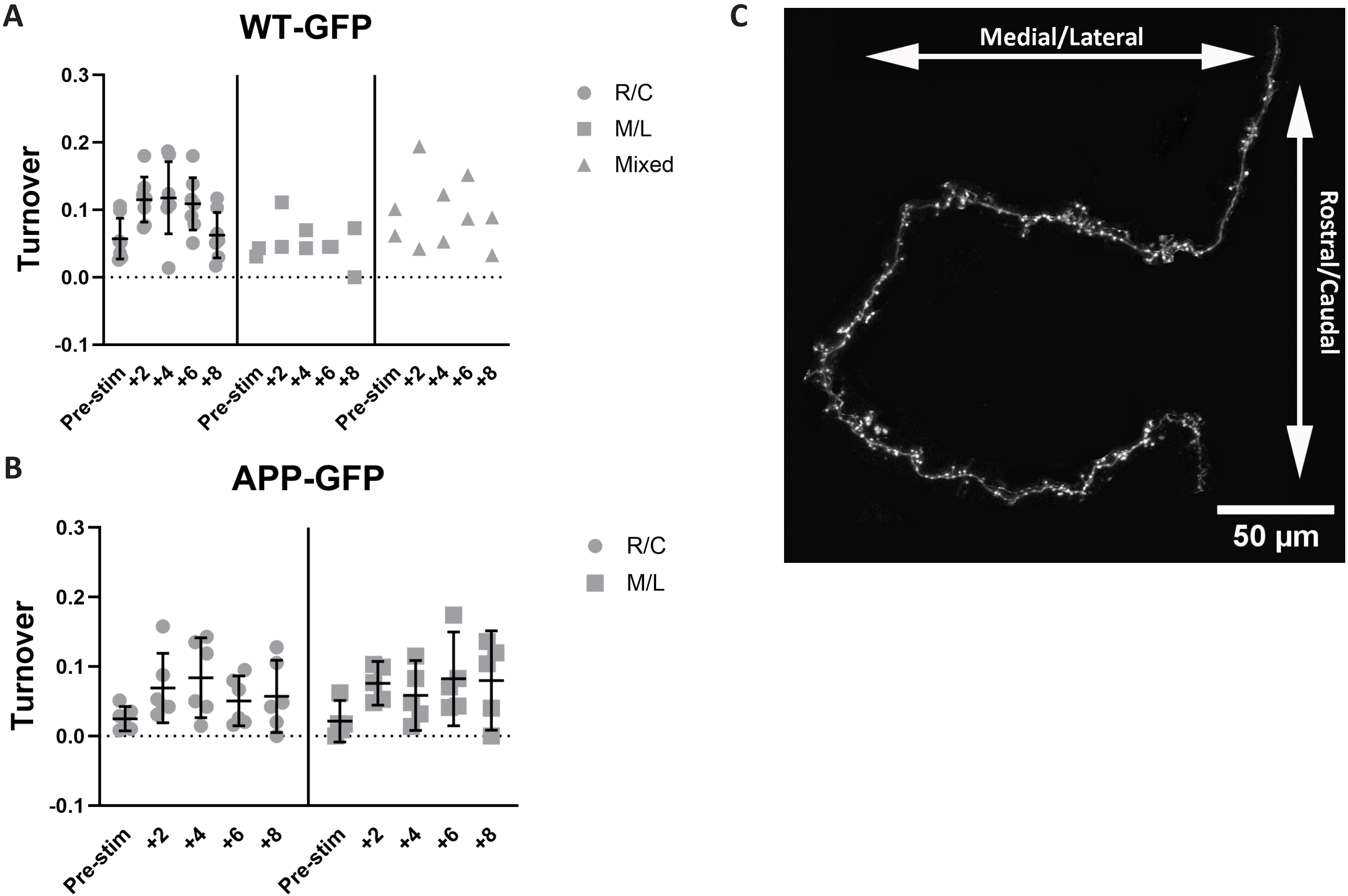

A
WT-GFP
Turnover
0.3
0.2
0.1
0.0
-0.1
Pre-stim
+2
+4
+6
+8
R/C
M/L
Mixed
B
APP-GFP
C
Medial/Lateral
Rostral/Caudal
50 µm

Supplementary figure 2. Orientation of imaged axons did not influence turnover of terminaux boutons. A&B) We classified imaged axons into 3 categories based on their orientation – rostral/caudal (RC), medial/lateral (ML) or mixed. We saw no trends suggesting differences in turnover based on orientation for either WT-GFP **(A)** or APP-GFP **(B)** groups. **C)** Representative example of a mixed orientation axon. (WT–GFP - wild type animal crossed onto Thy1-GFP background; APP-GFP – APP/PS1 animal crossed on Thy1-GFP background, error bars are 95% confidence intervals around the mean).
